# Development of Efficient Plant Regeneration and Transformation System for Impatiens Using *Agrobacterium tumefaciens *and Multiple Bud Cultures as Explants

**DOI:** 10.1186/1471-2229-10-165

**Published:** 2010-08-09

**Authors:** Yinghui Dan, Aaron Baxter, Song Zhang, Christopher J Pantazis, Richard E Veilleux

**Affiliations:** 1 Institute for Sustainable and Renewable Resources, Institute for Advanced Learning and Research, 150 Slayton Avenue, Danville, VA 24540, USA; 2 Department of Horticulture, Virginia Polytechnic Institute & State University, Blacksburg, VA 24061, USA; 3 Department of Forestry, Virginia Polytechnic Institute & State University, Blacksburg, VA 24061, USA; 4 Fisher Scientific, 3970 John's Creek Court, Suite 500, Suwanee, GA 30024, USA

## Abstract

**Background:**

Impatiens (*Impatiens walleriana*) is a top selling floriculture crop. The potential for genetic transformation of Impatiens to introduce novel flower colors or virus resistance has been limited by its general recalcitrance to tissue culture and transformation manipulations. We have established a regeneration and transformation system for Impatiens that provides new alternatives to genetic improvement of this crop.

**Results:**

In a first step towards the development of transgenic INSV-resistant *Impatiens*, we developed an efficient plant regeneration system using hypocotyl segments containing cotyledonary nodes as explants. With this regeneration system, 80% of explants produced an average of 32.3 elongated shoots per initial explant plated, with up to 167 elongated shoots produced per explant. Rooting efficiency was high, and 100% of shoots produced roots within 12 days under optimal conditions, allowing plant regeneration within approximately 8 weeks. Using this regeneration system, we developed an efficient *Agrobacterium*-mediated *Impatiens *transformation method using *in vitro *multiple bud cultures as explants and a binary plasmid (pHB2892) bearing *gfp *and *nptII *genes. Transgenic *Impatiens *plants, with a frequency up to 58.9%, were obtained within 12 to 16 weeks from inoculation to transfer of transgenic plants to soil. Transgenic plants were confirmed by Southern blot, phenotypic assays and T_1 _segregation analysis. Transgene expression was observed in leaves, stems, roots, flowers, and fruit. The transgenic plants were fertile and phenotypically normal.

**Conclusion:**

We report the development of a simple and efficient *Agrobacterium*-mediated transformation system for *Impatiens*. To the best of our knowledge, there have been no reports of *Agrobacterium*-mediated transformation of Impatiens with experimental evidence of stable integration of T-DNA and of *Agrobacterium*-mediated transformation method for plants using *in vitro *maintained multiple bud cultures as explants. This transformation system has the advantages of 1) efficient, simple and rapid regeneration and transformation (with no need for sterilization or a greenhouse to grow stock plants), 2) flexibility (available all the time) for *in vitro *manipulation, 3) uniform and desirable green tissue explants for both nuclear and plastid transformation using *Agrobacterium*-mediated and biolistics methods, 4) no somaclonal variation and 5) resolution of necrosis of *Agrobacterium*-inoculated tissues.

## Background

Impatiens is a top selling floriculture crop, with an annual value of approximately $155 million in the US reported in 2006 [1]. It grows throughout tropical Africa, India, southwest Asia, southern China, Japan, as well as parts of Europe, Russia, and North America [2], with *Impatiens walleriana *the most commonly cultivated species throughout the world [2].

Impatiens Necrotic Spot Virus (INSV) is a serious threat for the floriculture industry both in North America and in Europe [3-5]. Because no naturally INSV-resistant Impatiens cultivars have been available the possibility of transgenic virus resistance is attractive. This possibility depends, however, on the availability of a robust transformation system.

There are few reports in the literature dealing with *Impatiens *tissue culture and transformation. These include micropropagation [6-9], *in vitro *germination of immature ovules [10], growth of cotyledon sections [11], callus culture [12,13] and embryo and ovule culture [14,15]. The majority of these publications reported callus induction from explants using New Guinea, Java Impatiens and *Impatiens platypetala*, with no *de novo *plant regeneration. Plantlet production has only been reported via micropropagation.

In terms of *Impatiens *transformation reports, hairy root induction *in vivo *from cotyledons of *Impatiens balsamina *and *Impatiens hawkerii *was reported using *A. rhizogenes *[16,17]. GUS and PCR positive plants of *I. balsamina *were obtained from cotyledon explants using microprojectile bombardment [18]. A patent relating to *Agrobacterium*-mediated transformation of *Impatiens *was issued, but no experimental data confirming transformation were reported in the patent [19]. We report the development of a simple and efficient *Agrobacterium*-mediated transformation and regeneration system for *Impatiens *using *in vitro *maintained multiple bud cultures as transformation explants. To the best of our knowledge, there have been no reports of *Agrobacterium*-mediated transformation of Impatiens with experimental evidence of stable integration of T-DNA and of *Agrobacterium*-mediated transformation method for plants using *in vitro *maintained multiple bud cultures as explants.

## Results and discussion

### Regeneration protocol development

Cotyledonary node explants (CNE) were evaluated for shoot regeneration after 4 weeks on shoot induction media 1 (SIM1) and 2 (SIM2), supplemented with 5 μM 6-benzyladenine (BA) and 5 μM thidiazuron (TDZ), respectively. On SIM2 medium amended with TDZ, 43% of explants produced multiple shoots (with more than three shoots per single explant) whereas only 18% of explants formed multiple shoots on SIM1 containing BA. Once TDZ had been shown to promote greater shoot regeneration than BA from cotyledonary nodes of *Impatiens*, the concentration of TDZ in the regeneration medium was optimized. With *I. walleriana *cv. Accent Red, TDZ at 1, 3 and 7 μM had significantly (P < 0.05) greater frequencies (40.0%, 26.7% and 35.0%, respectively) of explants producing multiple shoots than 5 μM (6.7%), but there was no significant difference for percent of explants producing multiple shoots among the three concentrations (Table 1 ). For *I. walleriana *cv. Salmon Picote, the frequency (50.0%) of explants producing multiple shoots was significantly (P < 0.05) greater when using 1 μM TDZ than the other (from 10.0% to 26.7%) concentrations tested (Table 1).

**Table 1:** Mean percent of explants producing shoots of *I. walleriana *cvs. Accent Red and Salmon Picote after cotyledonary nodes were placed on medium with four different concentrations of TDZ 4 weeks after culture.

Cultivar	TDZ concentration (μM)	No. of explants	**% of explants producing shoots**^**x**^ (mean ± standard error)
Accent Red	1	20	40.0 ± 9.4 A
	3	30	26.7 ± 7.7 AB
	5	30	6.7 ± 7.7 B
	7	20	35.0 ± 9.4 A

Salmon Picote	1	26	50.0 ± 8.2 A
	3	30	10.0 ± 7.7 B
	5	30	26.7 ± 7.7 B
	7	22	18.2 ± 9.0 B

^x ^Treatments followed by different letters are significantly (P < 0.05) different using the LSD All-Pairwise Comparison Test (Statistix 9, Analytical Software, Tallahassee, FL, USA).

**Figure 1:**
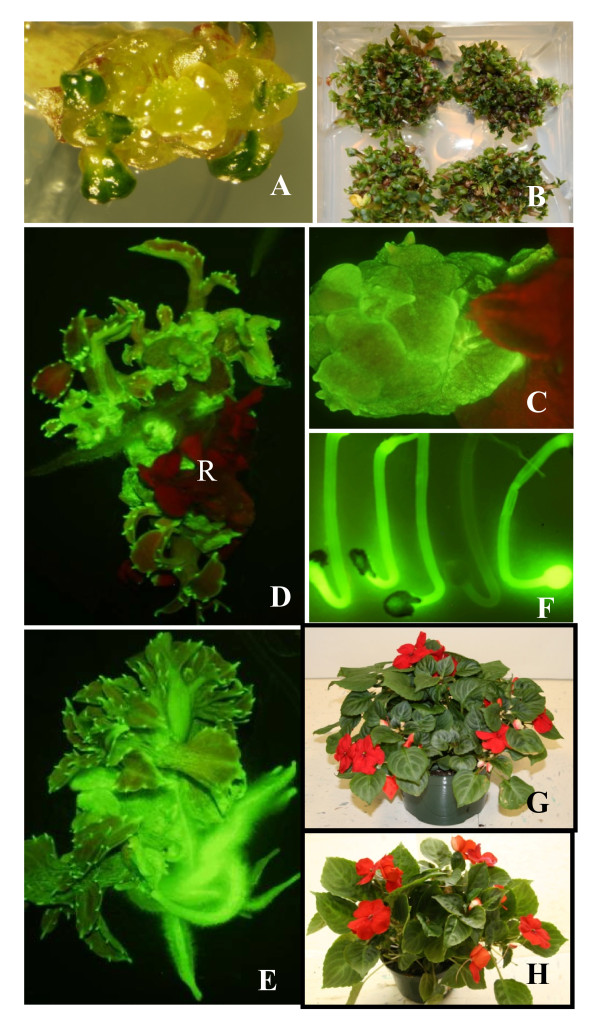
**Regeneration and transformation of *I. walleriana *cv. Accent Red**. Multiple bud induction 2 weeks after HSCCN explants were cultured on IM2 (A). Multiple shoot production approx. 6 weeks after HSCCN explants were initially cultured on IM2 (B). Transgenic buds expressing GFP produced approx. 3 weeks after selection on IM2 supplemented with 50 mg/l kanamycin and 250 mg/l carbenicillin (C). Transgenic shoots expressing GFP generated approx. 6 weeks after selection on IM2 supplemented with 50 mg/l kanamycin and 250 mg/l carbenicillin and nontransgenic shoots exhibiting dark red autofluorescence (pointed by R) of chlrorophll, indicating no GFP expression (D). Transgenic plantlets expressing GFP approx. 3 weeks after selection on IM4 supplemented with 50 mg/l kanamycin and 250 mg/l carbenicillin (E). GFP segregation in T_1 _plants (7 day-old seedlings) (F). A transgenic plant in soil (G). A wild type plant (non-transgenic plant) in soil (H).

To further optimize shoot regeneration frequency, we tested combinations of TDZ with BA at different concentrations using hypocotyl segments containing cotyledonary nodes (HSCCN). More than 95% of explants from both genotypes produced multiple shoots when using IM2 and IM3 with both BA and TDZ (data not shown), whereas TDZ at 1 μM without BA (IM1) promoted only 40% response for Accent Red and 50% response for Salmon Picote (Table 1). The combination of 2.3 μM TDZ and 1.8 μM BA (IM2 medium) generated significantly (P < 0.05) greater frequencies of explants (66.5% for Accent Red and 80.1% for Salmon Picote) producing massive shoots, determined as production of 15 to 167 elongated shoots from a single explant (an average of 32.3 elongated shoots per explants) compared to other treatments (Table 2). IM2 not only significantly increased the frequency of explants responding, but also increased number of shoots produced per explant and reduced time to produce the shoots. Multiple buds formed 2 to 3 weeks after culture (Figure 1A) and up to 167 shoots were produced from a single explant on IM2 (Figure 1B). Shoots were separated 3-4 weeks after multiple shoot induction on IM2 and transferred to shoot elongation medium IM4 similar to IM2 except with 0.23 μM TDZ and 0.22 μM BA for several weeks. For rooting, 95.8% to 100% of shoots produced roots within 2 to 3 weeks after the shoots were transferred to the rooting medium IM5. Kinetin at 139 μM produced the greatest number of shoots (8.5 shoots from the most responsive genotype) from shoot tips of an interspecific hybrid, T63-1, of a cross between *Impatiens platypetala *and a New Guinea *Impatiens *[6]. Han and Stephens [7] reported that BA at 10 μM was most effective for stimulating shoot multiplication using shoot tips as explants for the same hybrid, for which 9.3 shoots per explant were obtained. Also BA at 4 mg/dm^3 ^and kinetin at 12 or 20 mg/dm^3 ^induced multiple shoots from shoot tip explants of *I. walleriana *in micropropagation [9].

**Table 2:** Mean percent of explants of *I. walleriana *cvs. Accent Red and Salmon Picote producing multiple shoots from HSCCN 4 weeks after placement on media with three growth regulator combinations.

Cultivar	TDZ/BA concentration	No. of explants	**% of explants producing massive shoots/buds**^**x**^ (mean ± standard error)
Accent Red	1 μM TDZ + 0 μM BA (IM1)	121	20.5 ± 6.4 A
	2.3 μM TDZ + 1.8 μM BA (IM2)	121	66.5 ± 6.4 B
	1 μM TDZ + 2.2 μM BA (IM3)	119	42.6 ± 6.4 C

Salmon	1 μM TDZ + 0 μM BA (IM1)	65	59.5 ± 5.3 A
Picote	2.3 μM TDZ + 1.8 μM BA (IM2)	68	80.1 ± 4.6 B
	1 μM TDZ + 2.2 μM BA (IM3)	65	43.9 ± 5.3 A

^x ^Treatments followed by different letters are significantly (P < 0.05) different using the LSD All-Pairwise Comparison Test (Statistix 9, Analytical Software, Tallahassee, FL, USA).

Cotyledonary nodes have been the explants of choice for transformation of soybean and otherwise recalcitrant legumes [20-23]. They have recently been used to transform a member of the Bignoniaceae family [24]. Micropropagation of recalcitrant species of legumes, including common bean [25], grasspea [26], and *Sesbiana *[27], among others, has been facilitated by the use of cotyledonary nodes. Recently cothledonary nodes were identified as the only explants capable of regeneration for the ice plant, *Mesembranthemum crystallinum *[28]. TDZ was reported to induce a high frequency of plant regeneration in *Stachys sieboldii *[29], lingonberry [30], *Linum *[31], *Salicornia europaea *[32], *Cichorium intybus *[33], *Astragalus cicer *[34], *Carthamus tinctorius *[35], *Pisum sativum *[36] and ginger [37].

### Determination an optimal concentration of kanamycin for transformation selection

For shoot selection, the percentage of multiple bud cluster explants (MBCE) producing new growth was significantly (P < 0.05) lower when using kanamycin at 50 mg/l than at 0, 20 or 35 mg/l (18.2% vs. 100%, 91.5% and 86.4% for Accent Red and 15.7% vs. 100%, 80.6% and 83.3% for Salmon Picote. Kanamycin at 50 mg/l was chosen for shoot selection of both cultivars because it significantly (P < 0.05) inhibited shoot growth and this non-lethal concentration may allow recovery of transgenic plants having low expression of a neomycin phosphotransferase II (*NPTII*).

For root selection of Accent Red, kanamycin at 25, 35 and 50 mg/l significantly inhibited the percentages of shoots producing roots (22.2%, 11.1% and 0%, respectively) compared with no kanamycin. However, there was no significant difference among the percentages of shoots producing roots when using kanamycin at 25, 35 and 50 mg/l. Kanamycin at 35 mg/l was chosen for root selection because it prevented 90% of shoots from producing roots, and this non-lethal concentration may allow the recovery of transgenic plants having low *NPTII *expression.

### Effect of co-culture duration on *Agrobacterium *infection

After 6 and 7 days of co-culture a significantly (P < 0.05) greater percentage of explants (31.2% and 29.4%, respectively) exhibited a green fluorescent protein (GFP) expression compared to those receiving 1 or 2 days of co-culture when there was no GFP expression was observed and there were only a few shoots with GFP expression when MBCE were co-cultured for 3 or 5 days (data not shown). By using MBCE and co-culture of the explants on filter paper dampened with an inoculation medium, we were able to control *Agrobacterium *growth even 10 days after co-culture (data not shown). Cheng et al. [38,39] reported that placing explants on filter paper moistened with inoculation medium during co-culture significantly reduced *Agrobacterium *overgrowth compared to solid co-culture medium.

### Regeneration of transgenic plants

**Figure 2:**
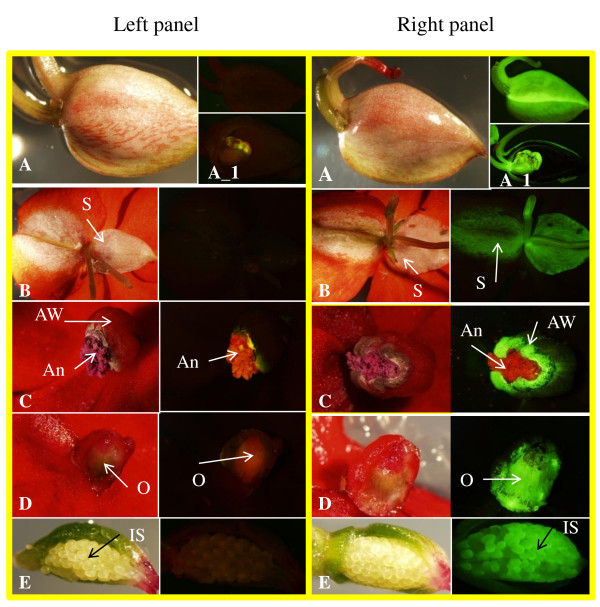
**GFP expression of transgenic plants of *I. walleriana *cv. Accent Red**. Left panel, Flower buds/organs and seeds of control plants viewed under tungsten light (left side) and UV light using GFP filters (right side); Right panel, Flower buds/organs and seeds of transgenic plants viewed under tungsten light (left side) and UV light using GFP filters (right side). A flower bud (A). Sectional slice of the flower bud (A_1). Posterior sepal (S) (B). Anthers (An) and anther wall (AW) (C). Sectional slice of an ovary (O) (D). Sectional slice of a seed pod with immature seeds (IS) (E).

Six to seven days after co-culture, MBCE were selected on IM2 supplemented with 50 mg/l kanamycin and 250 mg/l carbenicillin. Approximately 3 to 4 weeks after selection, GFP positive buds were observed (Figure 1C), with GFP positive shoots obtained 6 to 7 weeks after selection (Figure 1D). The percentage of explants producing GFP positive buds and/or shoots was 59%. GFP positive shoots were transferred to IM5 with 35 mg/l kanamycin and 250 mg/l carbenicillin to induce roots, and 79% of GFP positive shoots generated roots on IM5 2 to 4 weeks after culture (Figure 1E). Transgenic plants expressing GFP were transplanted into soil. The transformation cycle from inoculation to soil took approx. 2 to 3 months. The percentage of explants producing transgenic plants was 59% and the frequency of initial explants that produced independent transgenic plant events confirmed by Southern blot was 10.4%. Transgenic plants expressed GFP in many tissues, including leaf, stem, root, flower (including sepals, anther walls and ovaries), and immature seed (Figure 1 and 2). Transgenic plants appeared phenotypically normal and fertile (Figure 1G and 1H). Taha et al [18] reported that GUS and PCR positive shoots of *I. balsamina *were regenerated from bombarded cotyledon explants on MS medium supplemented with 1 mg/l BA and 75 mg/l hygromycin; subsequently, GUS and PCR positive plants were obtained on MS medium supplemented with 0.1 mg/l indole-3-acetic acid (IAA).

### PCR, Southern blot and R_1 _segregation analysis of transgenic *I. walleriana*

**Figure 3:**
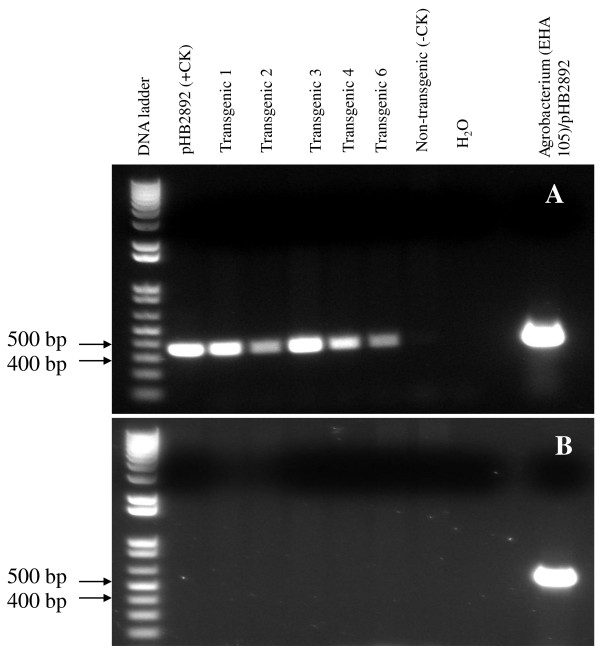
**PCR analysis of genomic DNA from five transgenic *I. walleriana *plants that expressed GFP using primers for the *nptII *gene (A) and the *picA *gene of *A. tumefaciens *chromosome (B)**.

Amplified PCR products corresponded to the expected size for the *nptII *gene in five GFP positive plants and in the *Agrobacterium*, but not in the wild type plant (non-transgenic plant) (Figure 3A). In addition, the PCR band for the *picA *gene was only present in the *Agrobacterium *strain EHA105 containing pHB2892 (Figure 3B). This indicated that the *nptII *gene had integrated into the genome of the transgenic plants, and was not due to *Agrobacterium *contamination of the transgenic plants.

**Figure 4:**
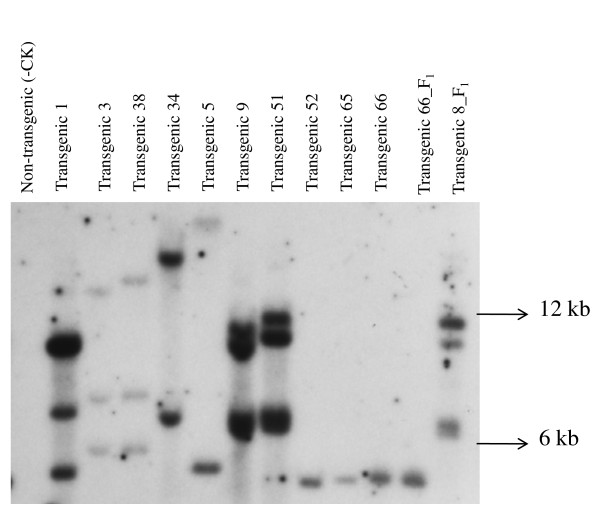
**Southern blot of genomic DNA from 12 transgenic plants that were GFP positive**. The DNA was digested with *EcoR1 *and the blots hybridized with an *nptII *gene probe. Transgenic plants 1, 3, 38, 34, 5, 9, 51, 52, 65 and 66 were T_0 _plants and transgenic plants 66_F_1 _and 8_F_1 _were T_1 _plants.

**Figure 5:**
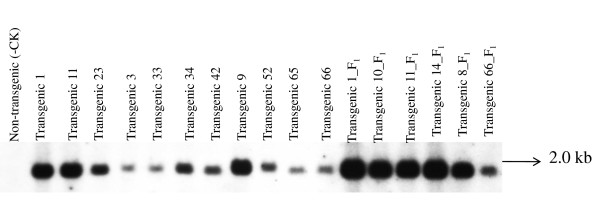
**Southern blot of genomic DNA from 17 transgenic plants that were GFP positive**. The DNA was digested with *HindIII *and the blots hybridized with a *gfp *probe. Transgenic plants 1, 11, 23, 3, 33, 34, 42, 9, 52, 65, 66 were T_0 _plants and transgenic plants 1_F_1_, 10_F_1_, 11_F_1_, 14_F_1_, 8_F_1 _and 66_F_1 _were T_1 _plants.

Up to twenty transgenic plants that expressed GFP were analyzed by Southern blot using probes of *nptII *and *gfp *genes. Southern blot hybridization with the *nptII *probe revealed the transgene copy number varying between one and three for ten T_0 _and two T_1 _transgenic plants (Figure 4). An expected band size of 1.9 kb, positioned within the T-DNA for the *gfp *gene, was present in eleven T_0 _and six T_1 _transgenic plants (Figure 5).

**Figure 6:**
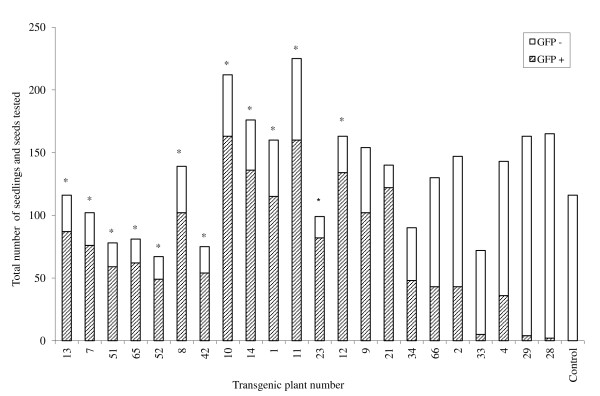
**Segregation for GFP expression in T_1 _progeny of 23 transgenic *I. walleriana *plants**. Control: non-transgenic plant. *Transgenic plant had a 3:1 ratio (P < 0.05) for segregation of GFP expression.

Segregation of GFP expression among the T_1 _plants for 13 transgenic T_0 _lines displayed the expected 3:1 ratio (p < 0.05) for a single gene hemizygous in the T_0 _(Figure 1F and 6). Segregation in the remaining ten transgenic exhibited a disproportionate number of nontransgenics in the T_1 _(Figure 6). No seedlings from the control *Impatiens *plant displayed GFP.

For our transformation system, GFP expression was used to select transformed buds and/or shoots 2 to 4 weeks after selection. The GFP positive buds and/or shoots were transferred to shoot elongation medium with 50 mg/l kanamycin for 3 to 4 weeks and the GFP positive shoots were transferred to rooting medium with 30 mg/l kanamycin for 2 to 4 weeks. Among selected GFP positive shoots tested, 100% of them produced transgenic plants that were confirmed by Southern blot and R_1 _segregation analysis. Among MBCE that were inoculated and plated on selection medium, 10% of them produced GFP positive buds and/or shoots. Approximately 40% to 94% of kanamycin resistant shoots have been reported to be non-transgenic (shoot escapes) when using kanamycin as a selective agent in many plant species [40]. For transformation of tomato cv. MicroTom, 91.5% of kanamycin resistant shoots after selection on 100 mg/l kanamycin were shoot escapes [41]. However, additional selection of kanamycin resistant shoots on rooting medium with 100 mg/l kanamycin yielded 95.6% transgenic plants [42]. An advantage of visualizing GFP expression in our system was to enable us to select transformation events at an early stage thus avoiding the transfer of non-transgenic shoot that survived the kanamycin selection, saving both time and labor.

## Conclusions

We report the development of a simple and efficient *Agrobacterium*-mediated transformation and regeneration system for *Impatiens*. To the best of our knowledge, there have been no reports of *Agrobacterium*-mediated transformation of *Impatiens *with experimental evidence of stable integration of T-DNA. Co-cultivation period, type of explants, a *gfp *gene as reporter marker to allow early stage selection of transformants and selection regime were critical factors for successful transformation of *Impatiens*.

Many *Agrobacterium*-mediated transformation methods have used explants derived directly from *in vivo *plants or from pre-cultured embryogenic calli for inoculation, due to ease of infection by *Agrobacterium*, but these methods have disadvantages such as expense of stock plant growth, detrimental sterilization procedures, time-consuming explant preparation, lack of flexibility, microbial contamination of explants, variation due to age and position of explants, all of which affect transformation efficiency. Our direct shoot regeneration system without an intervening callus phase is desirable for both nuclear and plastid transformation using *Agrobacterium*-mediated and biolistics methods for several reasons, including: 1) efficient, simple and rapid regeneration and transformation (with no need for sterilization or a greenhouse to grow stock plants), 2) flexibility (available all the time) for *in vitro *manipulation, 3) uniform and desirable green tissue explants for both nuclear and plastid transformation using *Agrobacterium*-mediated and biolistics methods and 4) no somaclonal variation. In addition, *Impatiens *CNE derived from *in vivo *seedlings necrosed, rotted and died several days after *Agrobacterium *inoculation [43]. Using *in vitro *MBCE derived from the HSCCN of *Impatiens *overcame this problem and enabled transgenic plant regeneration. Maziah *et al*. [44] inoculated a single bud isolated from *in vitro *multiple buds with *Agrobacterium *to produce transgenic banana. Our use of *in vitro *MBCE for *Agrobacterium *inoculation circumvented the problem of necrotic explants and resulted in more efficient transformation than inoculation of single buds. To our knowledge from literatures, there have been no reports of *Agrobacterium*-mediated transformation method for plants using *in vitro *maintained multiple bud cultures as explants.

A major challenge associated with plant transformation technology is the death of *Agrobacterium*-transformed tissues or cells, which severely limits the number of transgenic plants that can be regenerated, and the biotechnological exploitation of economically important crops. Hydrogen peroxide (H_2_O_2_) plays an important role in programmed cell death (PCD) and stress [45]. However, little is known about the molecular mechanisms regulating plant PCD, and the signaling pathway leading from H_2_O_2 _to the death of the tissues during transformation. *Impatiens *CNE derived from *in vivo *seedlings necrosed, rotted and died several days after *Agrobacterium *inoculation [43]. Using *in vitro *MBCE derived from the HSCCN of *Impatiens *overcame this problem and enabled transgenic plant regeneration. We can further investigate levels of cell death and the correlation between the cell death and H_2_O_2 _production of the both explant types and determine whether MBCE reduces the H_2_O_2 _production and subsequently it decreases cell death. A mechanistic knowledge of how the tissue death is triggered by the signaling molecule (H_2_O_2_) during the transformation is required in order to solve this issue.

Ornamental horticulture, and particularly floriculture, is well-suited to the application of genetic engineering technology because the product is not intended to be consumed, so commercialization may meet fewer obstacles without the need of food safety studies. However, genetic engineering technologies including transformation have had limited impact in ornamental horticulture. As the development of new varieties is an important driving force in the industry, there are, therefore, good prospects for the development of new genetically modified ornamental crops. Using the present protocol, thousands of transgenic Impatiens could be produced within 3 to 4 months. The transformation methodology developed in this study will thus be useful for future *Impatiens *improvement.

## Methods

### Materials

**Figure 7:**
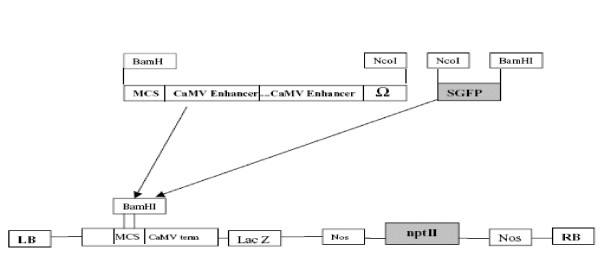
**The T-DNA region of pHB2892 plasmid with the *gfp *and *npt*II genes (47)**.

Seeds of *I. walleriana *cvs. Accent Red and Salmon Picote were obtained as a gift from Goldsmith Seed Co. (Gilroy, CA). Seeds were decontaminated by a gas sterilization method. Seeds were placed in a sterile 100 × 15 mm Petri dish. The Petri dish and a 250 ml beaker containing 100 ml Clorox™ bleach were placed in a 150 mm diameter desiccator, which had been surface sterilized with 70% alcohol. The desiccator was placed in a fume hood and 3.3 ml of 37% HCl (Sigma, reagent grade, 435570-2 (or 12 N HCl) were added drop by drop along the side of the beaker. The desiccator was placed under vacuum for 15 min, after which the valve was closed and the seeds left overnight. The next day the seeds were transferred to MAGENTA^® ^culture vessels (*Phyto*Technology Laboratories, Shawnee Mission, KS, USA.) containing approx. 100 ml germination medium (GM) medium (Table 3), a modified MS medium [46] and incubated at 24°C, 16 h photoperiod and light intensity of approx. 82.6 μmol for 12 to 20 days. *Agrobacterium *strains EHA105 and LBA 4404 containing plasmid pHB2892 [47] were used for transformation experiments. The plasmid pHB2892 contains a green fluorescent protein (*gfp*) gene driven by a double CaMV 35 S promoter, and a neomycin phosphotransferase II (*nptII*) gene driven by a *nos *promoter (Figure 7) [47].

**Table 3:** Components of germination medium and basal media for multiple shoot induction, shoot elongation and root induction media in *Impatiens walleriana*.

Medium component	Medium name and concentration (g/l)		
	
	Germination medium (GM)	Basal medium 1 for shoot induction (BM1)	Basal medium 2 for shoot induction (BM2)
MS Basal medium with vitamins (PhytoTechnology Laboratories, Product No: M519)	4.43	4.43	4.43
MES (Sigma, Cat. No.: M3671)	1.95	-	1.95
Sucrose (Fisher Scientific, Cat. No.: S5-12)	30	30	30
Agar (Sigma, Cat. No.: A7921)	8	7	6
pH	5.6	5.7	5.7

### Regeneration protocol development

Cotyledonary node explants (CNE) were excised from seedlings of *I. walleriana *cvs. Accent Red and Salmon Picote that had grown on solid MS basal medium for 8-12 days, prior to expansion of the first true leaves. The epicotyl was removed approx. 5 mm below the cotyledonary node, and the cotyledonary node was divided vertically producing two explants from each seedling. The cotyledonary node tissue was then wounded with a scalpel blade (Feather Safety Razor Co. LTD Medical Division, Japan) by making 8 to 10 vertical cuts. The CNE were subsequently cultured upright with the basal end embedded in 100 × 20 mm Petri dishes containing 25 ml of different media based on basal medium 1 (BM1) for shoot induction (Table 3) amended with 5 μM BA or TDZ at 1, 3, 5, or 7 μM for 4 weeks. Each Petri plate contained ten explants, each treatment was replicated four times and plates were arranged using a completely random design. All cultures were placed in a growth chamber (Enconair, Ecological Chambers, Inc., Manitoba, Canada) at 25°C with 16 h photoperiod.

To improve regeneration efficiency and simplify explant preparation, we prepared hypocotyl segments containing cotyledonary nodes (HSCCN) from seedlings by removing epicotyls just above cotyledon nodes, both cotyledons and hypocotyls at a position approximately 0.5 cm below the cotyledonary node. The hypocotyl segments were placed upright, with the basal end embedded in 100 × 25 mm Petri dishes containing approx. 40 ml of any of three different shoot induction media (IM1, IM2, and IM3). IM1, IM2 and IM3 contained basal medium 2 (Table 3), supplemented with 1 μM TDZ (IM1), 2.3 μM TDZ and 1.8 μM BA (IM2) or 1 μM TDZ and 2.2 μM BA (IM3), respectively. After 3-4 weeks on the media, multiple buds or shoots were transferred to 100 × 25 mm Petri dishes containing approx. 40 ml of shoot elongation medium (IM4), which had basal medium 2 plus 0.05 mg/l TDZ and 0.05 mg/l BA. Several weeks after shoot elongation, shoots were transferred to 100 × 25 mm Petri dishes containing approx. 40 ml rooting medium (IM5), which had basal medium 2 plus 0.5 mg/l indole-3-butyric acid (IBA), for 2-3 weeks. All cultures were placed in a growth chamber (Enconair, Ecological Chambers, Inc., Manitoba, Canada) under 24°C, 16 h photoperiod and light intensity of approximately 82.6 μmol. All experiments were conducted two to four times independently.

### Determination of optimal concentration of kanamycin for transformation selection

To determine the optimal kanamycin concentration for shoot selection, we used MBCE approx. 0.5 cm diam isolated from 2-7 month old cultures that had been subcultured every month on IM2, and placed them on fresh IM2 with 0, 20, 35 and 50 mg/l kanamycin for 6 to 7 weeks. After this treatment, the cultures were examined for evidence of new growth (alive) or not (dead or necrotic). For root selection, individual shoots were separated from multiple shoot clusters and cultured on IM5 supplemented with 0, 25, 35 and 50 mg/l kanamycin for approximately 4 weeks, after which they were examined for evidence of root growth. Explants were grown under conditions described previously. All experiments were conducted three times independently.

### *Agrobacterium *preparation and transformation

*Agrobacterium *strains were grown according to Dan *et al*. [42], except with 50 mg/l kanamycin in LB medium. The bacteria were suspended in liquid medium containing 2.22 g/l MS basal medium, 30 g/l sucrose and 0.2 mM acetosyringone (AS) with pH of 5.4. Multiple bud cluster explants (MBCE) approx. 0.5 cm in diameter were isolated from two to seven month old cultures that were sub-cultured every month on IM2. The MBCE were inoculated in 30 ml *Agrobacterium *solution at OD_600 _= 0.5 for 30 min at room temperature after which the *Agrobacterium *solution was removed with a sterile pipette. A modification to the method of Cheng *et al*. [39] was used, where the explants were co-cultured in a sterile 100 × 15 mm Petri dishes Petri dish containing a filter paper (Whatman 4) soaked with 40 μl inoculation medium at 24°C in the dark for 1 to 7 days. After co-culture, the explants were cultured on IM2 supplemented with 50 mg/l kanamycin and 250 mg/l carbenicillin for multiple shoot induction. Multiple shoots that formed 5-8 weeks after selection were transferred onto IM4 plus 50 mg/l kanamycin and 250 mg/l carbenicillin for shoot elongation. Two to three weeks after shoot elongation, individual shoots were separated and transferred into IM5 with 35 mg/l kanamycin and 250 mg/l carbenicillin for rooting. All culture vessels and conditions were as described above. All experiments were conducted two or three times independently. An independent transgenic plant event was determined as only one transgenic plant that had regenerated from a single initial explant to ensure that each regenerant represented an independent transformation when multiple transgenic plants were obtained from a single initial explant. The transgenic plants were transplanted to soilless mix (Product No.: 74364300, Microcle-Gro Lawn Product, Inc., Marysville, OH, USA) in 15 cm diam pots and acclimated to *in vivo *conditions by placing each pot in a plastic bag for 2 weeks and then removing the plastic bag. The pots were placed in a growth chamber (Enconair, Ecological Chambers, Inc., Manitoba, Canada) under 20°C, 11 h photoperiod and light intensity of approximately 40 μmol where they were hand pollinated.

### PCR, Southern blot and R_1 _segregation analysis

Plant genomic DNA for PCR was extracted using the Qiagen DNeasy Plant Mini Kit (Cat#69104, Maryland, USA). Primers used for the *gfp *gene were 5'-AGGGCGATGCCACCTA-3' and 5'-GACTGGGTGCTCAGGTA-3'. Primers for the *nptII *gene were 5'-TGTGCTCGACGTTGTCACTGAA-3' and 5'-CACCATGATATTCGGCAAGCAG-3'. Primers for the *picA *gene (plant-inducible chromosomal gene A) of *A. tumefaciens *chromosome were 5'-ATGCGCATGAGG CTCGTCTTCGAG-3' and 5' GACGCAACGCATCCTCGATCAGCT-3'. PCR was conducted in a total volume of 20 μl, containing 100 ng template DNA, 2 μl 10× buffer, 1 μl 4 mM dNTP, 0.2 μl of 50 μM primers, 0.2 μl of 5000 U/ml Taq DNA polymerase (NEB, Maryland, USA) with a MyCycler Thermal Cycler (Bio-Rad, USA). The PCR conditions were: initial denaturation at 95°C for 5 min, 30 cycles of denaturation at 95°C for 30 sec, annealing at 49°C for 30 sec for the *gfp *gene, 62°C for the *nptII *gene or 70°C for the *picA *gene, and extension at 72°C for 30 sec, followed by final extension at 72°C for 5 min and holding at 4°C.

Southern blot analysis was performed by Lofstrand Labs Ltd. (Gaithersburg, MD 20879). Approx. 15 μg genomic DNA of Impatiens plants were digested with *EcoR1 *for the *nptII *probe or *Hind III *for the *gfp *probe. The digested DNA samples were loaded onto a 350 ml 0.7% TBE agarose gel and the gel was electrophoresed at 50 V for 18 h. It was transferred to a nylon membrane Nytran Supercharge (Whatman/Schleicher and Schuell). Each membrane was UV linked and air-dried. The membrane was prehybridized using 6× SSC, 5× Denhardt's solution, and 0.5% SDS at 68°C for 6 h. They were separately hybridized using the random primed probes of *nptII *and *gfp *template DNA that were labeled with P^32^. The hybridizations were carried out at 68°C for 27 h. The membranes was washed in 2× SSC+0.1%SDS at 68°C with three buffer changes over a period of 60 min; and 20 min at 68°C with 0.1× SSC + 0.1% SDS. The membranes were autoradiographed for approximately 41 h and 4 days using an intensifier screen at -80°C for the *nptII *and *gfp *genes, respectively.

Approximately 100 seeds of each transgenic plant and a wild type *Impatiens *as control were geminated on moistened filter paper in 100 × 15 mm Petri plates sealed with parafilm and incubated at 25°C in the dark for 7 days. The seedlings and some non-geminated seeds were screened under an Olympus SZX12 stereomicroscope with a GFP filter for the presence of GFP (Figure 4f). The number of GFP positive and negative seedlings and the number of GFP positive and negative seeds were recorded. Chi-square analysis was conducted to determine goodness of fit to a 3:1 ratio of GFP positive to GFP negative seedlings and seeds.

## Authors' contributions

YD performed transformation and regeneration improvement experiments, data analysis, drafted and revised the manuscript and supervised the study as a principal investigator. AB performed experiments for initial regeneration method development as part of his MS thesis. SZ performed PCR and Impatiens DNA isolation. CP performed GFP assay for F1 seedlings, analyzed its GFP expression data and technical assistance. RV directed AB in graduate studies, provided technical and partial financial support and revised the manuscript critically. All authors read and approved the final manuscript.

## References

[B1] USDA Floriculture Crops, NASS 2006

[B2] Grey-Wilson C Impatiens of Africa: morphology, pollination and pollinators, ecology, phytogeography, hybridisation, keys and a systematic treatment of all African species: with a note on collecting and cultivation 1980 Balkema, Rotterdam: A. A.

[B3] Law MD Moyer JW A tomato spotted wilt-like virus with a serologically distinct N-protein J Genl Virol 1990 71 933 938 10.1099/0022-1317-71-4-933

[B4] Deangelis JD Sether DM Rossignol PA Transmission of impatiens necrotic spot virus in peppermint by western flower thrips (Thysanoptera, Thripidae) J Econ Entomol 1994 87 197 201

[B5] Vaira AM Roggero P Luisoni E Masenga V Milne RG Lisa V Characterization of 2 Tospoviruses in Italy - Tomato Spotted Wilt and Impatiens Necrotic Spot Plant Pathol 1993 42 530 542 10.1111/j.1365-3059.1993.tb01533.x

[B6] Stephens LC Krell SL Weigle JL In vitro-propagation of Java, New-Guinea, and Java-x-New-Guinea Impatiens Hortscience 1985 20 362 363

[B7] Han K Stephens LC Growth-regulators affect in vitro-propagation of 2 interspecific *Impatiens *hybrids Sci Hort 1987 32 307 313 10.1016/0304-4238(87)90096-3

[B8] Nikolova RV Lall N Bosa AJN An assessment of the conditions for the rapid propagation of *Impatiens flanaganae *in-vivo and in-vitro Acta Hortic 1996 440 633 638

[B9] Witomska M Lukaszewska A Effect of cytokinin concentration and explant type on micropropagation of *Impatiens *× *walleriana *Hook Annals of Warsaw Agricultural University, Horticulture (Landscape Architecture) 2003 24 35 40

[B10] Han KC Stephens LC Carbohydrate and nitrogen-sources affect respectively in vitro germination of immature ovules and early seedling growth of *Impatiens platypetala *Lindl Plant Cell Tiss Org Cult 1992 31 211 214

[B11] Han KC In-vitro shoot regeneration from cotyledons of immature ovules of *Impatiens platypetala *Lindl In Vitro Cell Dev Biol-Plant 1994 30P 108 112

[B12] He YK Xi T Relationship between the accumulation of anthocyanin and phenylalanine ammonia lyase in *Impatiens balsamina* Plant Physiol Commun 1989 2 35 38

[B13] Josekutty PC Mpikeleli P Nikolova RV Calcium mediated callus induction in *Impatiens flanaganiae* Phyton-Int J Exp Bot 1998 63 A 199

[B14] Arisumi T In vitro culture of embryos and ovules of certain incompatible selfs and crosses among *Impatiens *species J Am Soc Hort Sci 1980 105 629 631

[B15] Arisumi T Rescuing abortive Impatiens hybrids through aseptic culture of ovules J Am Soc Hort Sci 1985 110 273 276

[B16] Xiang TH Wang LL Pang JL Chen M Xu C Hairy root induced by wild-type *Agrobacterium rhizogenes *K599 in soybean, cucumber and garden balsam *in vivo* Hereditas 2005 27 783 786 16257909

[B17] Milosevic S Subotic A Cingel A Jevremovic S Ninkovic S Efficient genetic transformation of *Impatiens hawkerii *Bull. (Balsamiaceae) Using *Agrobacterium rhizogenes* Arch Biol Sci 2009 61 467 474 10.2298/ABS0903467M

[B18] Taha AM Wagiran A Ghazali H Huyop F Parveez GKA Optimization and transformation of garden balsam, *Impatiens balsamina*, mediated by microprojectile bombardment Biotechnology 2009 8 1 12 10.3923/biotech.2009.44.52

[B19] Chou TS Production of transgenic impatiens United States Patent No. 6,121,511 2000

[B20] Paz MM Shou HX Guo ZB Zhang ZY Banerjee AK Wang K Assessment of conditions affecting *Agrobacterium*-mediated soybean transformation using the cotyledonary node explant Euphytica 2004 136 167 179 10.1023/B:EUPH.0000030670.36730.a4

[B21] Anuradha TS Jami SK Datla RS Kirti PB Genetic transformation of peanut (*Arachis hypogaea *L.) using cotyledonary node as explant and a promoterless *gus::nptII *fusion gene based vector J Biosci 2006 31 235 246 10.1007/BF02703916 16809856

[B22] Xue RG Xie HF Zhang B A multi-needle-assisted transformation of soybean cotyledonary node cells Biotechnol Lett 2006 28 1551 1557 10.1007/s10529-006-9123-6 16937246

[B23] Muruganantham M Amutha S Selvaraj N Vengadesan G Ganapathi A Efficient *Agrobacterium*-mediated transformation of *Vigna mungo *using immature cotyledonary-node explants and phosphinothricin as the selection agent In Vitro Cell Dev Biol-Plant 2007 43 550 557 10.1007/s11627-007-9060-7

[B24] Aslam M Singh R Anandhan S Pande V Ahmed Z Development of a transformation protocol for *Tecomella undulata *(Smith) Seem from cotyledonary node explants Sci Hort 2009 121 119 121 10.1016/j.scienta.2009.01.007

[B25] Dang W Wei ZM High frequency plant regeneration from the cotyledonary node of common bean Biol Plant 2009 53 312 316 10.1007/s10535-009-0056-5

[B26] Barik DP Naik SK Mohapatra U Chand PK High-frequency plant regeneration by in vitro shoot proliferation in cotyledonary node explants of grasspea (*Lathyrus sativus *L.) In Vitro Cell Dev Biol-Plant 2004 40 467 470 10.1079/IVP2004549

[B27] Jha AK Prakash S Jain N Nanda K Gupta SC Micropropagation of *Sesbania rostrata *from the cotyledonary node Biol Plant 2004 48 289 292 10.1023/B:BIOP.0000033458.88441.67

[B28] Sunagawa H Agarie S Umemoto M Makishi Y Nose A Effect of urea-type cytokinins on the adventitious shoots regeneration from cotyledonary node explant in the common ice plant, *Mesembryanthemum crystallinum* Plant Production Science 2007 10 47 56 10.1626/pps.10.47

[B29] Li W Dong W Zhao Dl Guo GQ Zheng GC TDZ induced high frequency of plantlet regeneration from axillary node of *Stachys sieboldii* Xibei Zhiwu Xuebao 2002 22 965 969

[B30] Debnath SC A two-step procedure for adventitious shoot regeneration from in vitro-derived lingonberry leaves: Shoot induction with TDZ and shoot elongation using zeatin Hortscience 2005 40 189 192

[B31] Mundhara R Rashid A TDZ-induced triple-response and shoot formation on intact seedlings of Linum, putative role of ethylene in regeneration Plant Sci 2006 170 185 190 10.1016/j.plantsci.2005.06.015

[B32] Shi XL Han HP Shi WL Li YX NaCl and TDZ are two key factors for the improvement of In vitro regeneration rate of Salicornia europaea L J Integr Plant Biol 2006 48 1185 1189 10.1111/j.1744-7909.2006.00342.x

[B33] Yucesan B Turker AU Gurel E TDZ-induced high frequency plant regeneration through multiple shoot formation in witloof chicory (Cichorium intybus L.) Plant Cell Tiss Org Cult 2007 91 243 250 10.1007/s11240-007-9290-8

[B34] Basalma D Uranbey S Gurlek D Ozcan S TDZ-induced plant regeneration in Astragalus cicer L Afr J Biotechnol 2008 7 955 959

[B35] Basalma D Uranbey S Mirici S Kolsarici O TDZ × IBA induced shoot regeneration from cotyledonary leaves and in vitro multiplication in safflower (Carthamus tinctorius L.) Afr J Biotechnol 2008 7 960 966

[B36] Zhihui S Tzitzikas M Raemakers K Zhengqiang M Visser R Effect of TDZ on plant regeneration from mature seeds in pea (Pisum sativum) In Vitro Cell Dev Biol-Plant 2009 45 776 782 10.1007/s11627-009-9212-z

[B37] Lincy A Sasikumar B Enhanced adventitious shoot regeneration from aerial stem explants of ginger using TDZ and its histological studies Turk J Bot 2010 34 21 29

[B38] Cheng M Hu T Layton J Liu CN Fry JE Desiccation of plant tissues post-*Agrobacterium *infection enhances T-DNA delivery and increases stable transformation efficiency in wheat In Vitro Cell Dev Biol-Plant 2003 39 595 604 10.1079/IVP2003471

[B39] Cheng M Lowe BA Spencer TM Ye X Armstrong CL Factors influencing *Agrobacterium*-mediated transformation of monocotyledonous species In Vitro Cell Dev Biol-Plant 2004 40 31 45 10.1079/IVP2003501

[B40] Dan Y Biological Functions of Antioxidants in Plant Transformation In Vitro Cell Dev Biol-Plant 2008 44 149 161 10.1007/s11627-008-9110-9

[B41] Dan Y Armstrong CL Dong J Feng X Fry JE Keithly GE Martinell BJ Roberts GA Smith LA Tan L Duncan DR Lipoic Acid----A Unique Plant Transformation Enhancer In Vitro Cell Dev Biol-Plant 2009 45 630 638 10.1007/s11627-009-9227-5

[B42] Dan Y Yan H Munyikwa T Dong J Zhang Y Armstrong CL MicroTom---A high-throughput model transformation system for functional genomics Plant Cell Rep 2006 25 432 44 10.1007/s00299-005-0084-3 16341726

[B43] Baxter A Regeneration and transformation of *impatiens walleriana *using cotyledonary node culture 2005 Virginia Polytechnic Institute and State University, Blacksburg, MS Thesis

[B44] Maziah M Sariah M Sreeramanan S Transgenic banana Rastali (AAB) with β-1, 3-glucanase gene for tolerance to Fusarium wilt race 1 disease via *Agrobacterium*-mediated transformation system Plant Pathol J 2007 6 271 282 10.3923/ppj.2007.271.282

[B45] Gechev ST Jacques Hille J Hydrogen peroxide as a signal controlling plant programmed cell death J Cell Biol 2005 168 17 20 10.1083/jcb.200409170 15631987 PMC2171664

[B46] Murashige T Skoog F A revised medium for rapid growth and bio assays with tobacco tissue cultures Physiol Plant 1962 15 473 497 10.1111/j.1399-3054.1962.tb08052.x

[B47] Molinier J Himber C Hahne G Use of green fluorescent protein for detection of transformed shoots and homozygous offspring Plant Cell Rep 2000 19 219 223 10.1007/s002990050002 30754898

